# Different transcriptional responses by the CRISPRa system in distinct types of heterochromatin in *Drosophila melanogaster*

**DOI:** 10.1038/s41598-022-15944-7

**Published:** 2022-07-09

**Authors:** Andrea Ortega-Yáñez, Samantha Cruz-Ruiz, Martha Vázquez, Mario Zurita

**Affiliations:** Departamento de Genética del Desarrollo Y Fisiología Molecular, Instituto de Biotecnología, Universidad Nacional Autónoma de México, Av. Universidad 2001, Cuernavaca, Morelos 62250 México

**Keywords:** Developmental biology, Genetics, Molecular biology

## Abstract

Transcription factors (TFs) activate gene expression by binding to elements close to promoters or enhancers. Some TFs can bind to heterochromatic regions to initiate gene activation, suggesting that if a TF is able to bind to any type of heterochromatin, it can activate transcription. To investigate this possibility, we used the CRISPRa system based on dCas9-VPR as an artificial TF in *Drosophila*. dCas9-VPR was targeted to the *TAHRE* telomeric element, an example of constitutive heterochromatin, and to promoters and enhancers of the HOX *Ultrabithorax* (*Ubx*) and *Sex Combs Reduced* (*Scr*) genes in the context of facultative heterochromatin. dCas9-VPR robustly activated *TAHRE* transcription, showing that although this element is heterochromatic, dCas9-VPR was sufficient to activate its expression. In the case of HOX gene promoters, although Polycomb complexes epigenetically silence these genes, both were ectopically activated. When the artificial TF was directed to enhancers, we found that the expression pattern was different compared to the effect on the promoters. In the case of the *Scr* upstream enhancer, dCas9-VPR activated the gene ectopically but with less expressivity; however, ectopic activation also occurred in different cells. In the case of the *bxI* enhancer located in the third intron of *Ubx*, the presence of dCas9-VPR is capable of increasing transcription initiation while simultaneously blocking transcription elongation, generating a lack of functional phenotype. Our results show that CRISPRa system is able to activate transcription in any type of heterochromatin; nevertheless, its effect on transcription is subject to the intrinsic characteristics of each gene or regulatory element.

## Introduction

Gene expression mediated by RNA polymerase II (RNPII) in eukaryotic cells is modulated by the action of activators that recruit the basal transcription machinery to the promoter to form the preinitiation complex (PIC)^1,2^. In some cases, such as the transcriptional activation mediated by nuclear hormone receptors, the GAGA factor and the heat shock factor, the transcriptional activator binding site could be located near the promoter^3–5^. However, in metazoans, most of the developmentally regulated genes, as well as genes that respond to different signal transduction pathways, are activated by the action of enhancers^6,7^. These cis-regulatory elements (CREs) are recognized by multiple transcription factors (TFs) and are able to operate at large distances from the promoter by the formation of chromatin loops through the interaction between transcription factors and components of PIC^8–10^. In general, the interaction of the TF with coactivators and/or with elements of the PIC occurs through a specific region known as the activation domain^11^. Transcriptional activators recruit protein complexes that modify and/or remodel chromatin to maintain transcriptional permissive regions known as euchromatin^12–14^.

On the other hand, in nontranscribed regions, the chromatin conformation, called heterochromatin, is highly compacted into a “closed stage”^15^. In general, two types of heterochromatin have been identified based on the degree of compaction, which is linked to specific histone modifications. Facultative heterochromatin is present in regions that include silenced genes in specific cell types, and its promoters are enriched with the repressive mark tri-methylation of lysine 27 of histone 3 (H3K27me3)^16,17^. This histone modification is introduced by components of the Polycomb group of genes in *Drosophila*, which include Polycomb Repression complexes 1 and 2 (PRC1, PRC2) through the action of the methyltransferase Enhancer of Zeste [*E(z*)]^18^. The action of PRC1 and PRC2 ensures the epigenetic silencing of genes during development^19^. In contrast, constitutive heterochromatin, considered to have a higher state of compaction of chromatin, is mostly present in telomeric and centromeric sequences, and it is characterized by enrichment of the trimethylation of lysine 9 of the histone 3 (H3K9me3) mark^20^.

It has been established that the conformation of chromatin is a determinant in the activation of gene expression. However, many TFs, mostly pioneers, recognize specific DNA elements in compact chromatin and are able to alter the structure of the nucleosome and recruit factors to activate transcription and open the chromatin^14^. It seems that this is the first event in transcription activation, suggesting that sending a transcriptional activator to any region in the chromatin could potentially cause its transcriptional activation.

The CRISPR/Cas9 system has been modified either to activate (CRISPRa) or to repress (CRISPRi) transcription^21^. To induce the transcription of specific genes, a dead Cas9 (dCas9) has been fused to the activation domains of several TFs, with dCas9-VPR being one of the most widely used^22^. VPR is comprised of three activation domains derived from VP6, p65 and Rta TFs, and it has been used to activate transcription in a variety of models^23^. The dCas9 fused to activator domains is usually directed upstream of the transcription start site (TSS) of a specific gene, and recently, it has been used to activate CREs such as enhancers^24,25^.

Based on this information, our main question was whether the dCas9-VPR system could activate transcription in any type of heterochromatin in a complete organism. For this purpose, we used *Drosophila* as a model organism since the combination of the GAL4-*UAS* system with CRISPRa-dCas9-VPR allows precise genetic manipulation in a complete animal^26^. To test this system in constitutive heterochromatin, we analyzed whether the telomeric *TAHRE* element, which is maintained as silenced constitutive heterochromatin, can be activated by dCas9-VPR. For the activation of facultative heterochromatin, we selected the HOX genes *Ultrabithorax* (*Ubx*) and *Sex combs reduced* (*Scr*). These genes are highly regulated during fly development, and their regulated silencing is maintained by PRC1 and PRC2^27^.

Intriguingly, dCas9-VPR was able to transcribe both types of heterochromatic elements during development. In the telomeric element, transcription was efficiently activated, showing that dCas9-VPR can act as a pioneer TF. In the case of HOX genes, dCas9-VPR was able to activate transcription; however, the effect on the activation of transcription was different between enhancers and promoters. In particular, the occupation of the synthetic transcriptional activator in the *bxI* enhancer located in a *Ubx* intron increases transcription initiation, but at the same time, it seems to block the passage of RNPII, generating a loss-of-function *Ubx* phenotype. These results show that the activation of transcription in different types of heterochromatin by the dCas9-VPR system generates distinct transcriptional responses that depend on their location and the roles they may have in the activation of transcription.

## Results

### Ectopic transcriptional activation of telomeric sequences in *D. melanogaster* by the CRISPRa system

**Figure 1:**
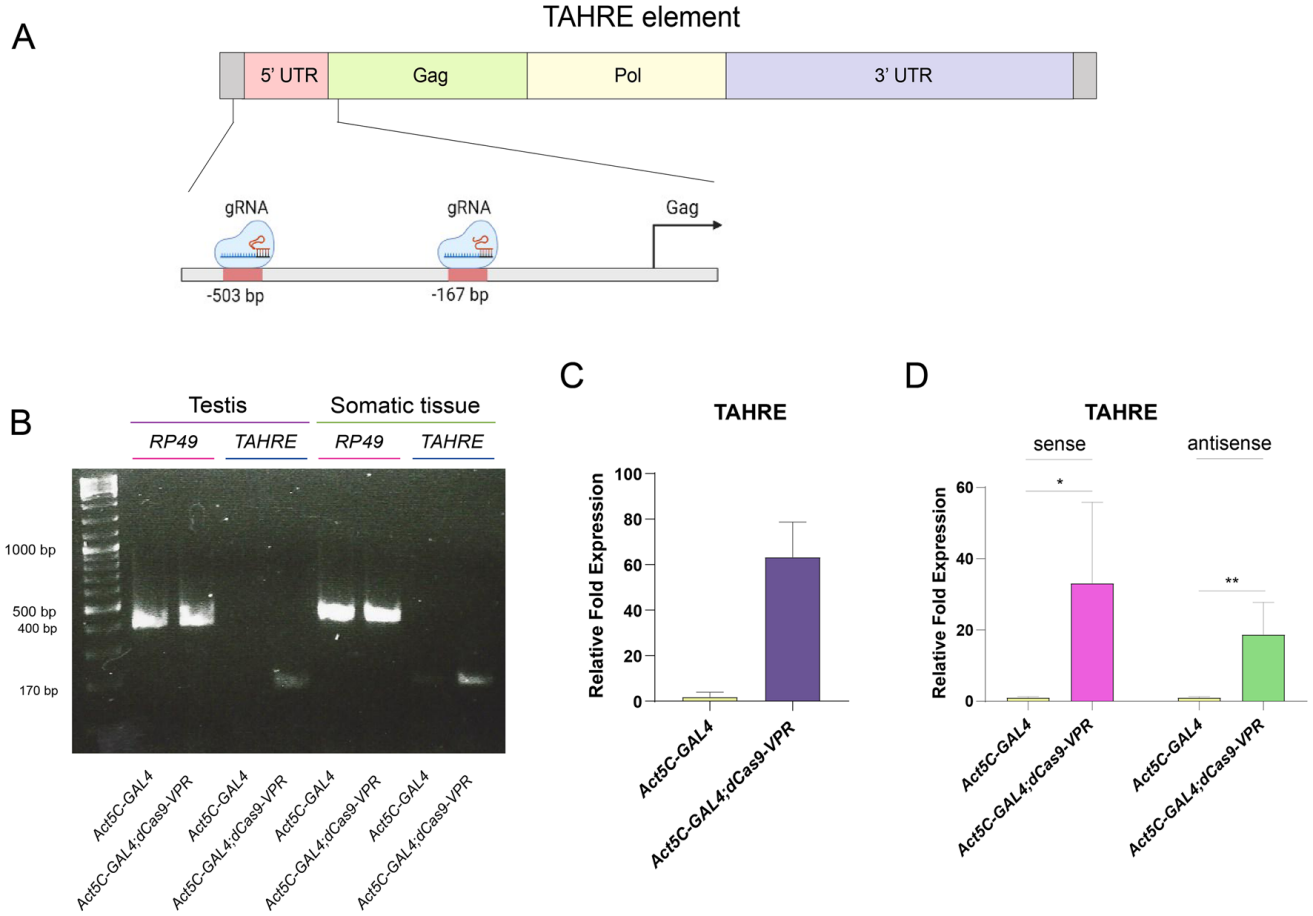
Transcription activation of the heterochromatic element *TAHRE* mediated by the dCas9-VPR. (**A**) Representation of the *TAHRE* element present in subtelomeric regions and the location of the designed gRNAs. The position of the gRNAs are indicated in Supp. Table 1. (**B**) Expression of *TAHRE* in somatic tissue in adult males (carcasses without testis) and in testis evaluated by RT-PCR. *Act5C-GAL4;UAS-dCas9-VPR* indicates the RT-PCR from total RNA from flies in which the dCas9 is directed to the *TAHRE* 5´region compared to the *Act5C-GAL4 * control flies. *rp49* mRNA was used as a loading control. (**C**) Transcript accumulation of *TAHRE* RNA in adult testis evaluated by RT-qPCR experiments from two biological replicates. Transcript levels of  *rp49*  were used as a reference. (**D**) *TAHRE* sense and antisence transcripts accumulation. Strand specific RT-qPCR was performed using strand specific primers for the cDNA synthesis (Sup. Table 1).

In *Drosophila,* telomeres are maintained by the transposition of *non-long terminal repeat* (*non-LTR*) retrotransposons. These retro-transposable elements (rTE), also referred as the *HTT* array, are known as *HeT-A*, *TART* and *TAHRE*^28^. These elements are silenced via the Piwi-piRNA pathway, which requires the transcription of segments of the rTE during oogenesis to induce the formation of heterochromatin^29^. We chose this region to test the transcription of constitutive heterochromatin using the CRISPRa system. We designed double single guides RNAs (gRNAs) directed upstream of the 5´ region of the *TAHRE* element, and one of the designed gRNA is also directed to the 3’ region of this rTE (Fig. 1A, Sup. Table 1). *A UAS-dCas9-VPR* transgene was expressed in all cell types and developmental stages under the control of the GAL4*-*UAS system using the *Act5C-GAL4* driver (Fig. 1A). We used the *Act5C* driver since it is not strong; therefore, the levels of dCas9-VPR are not high, similar to the native levels of most TFs.

Intriguingly, we did not detect any phenotype in these flies during development. However, we analyzed the ectopic expression of the *TAHRE* element by standard semiquantitative RT–PCR and RT–qPCR in adults (Sup. Table 2). We observed that in adult flies with the CRISPRa genotype, the expression of *TAHRE* was induced in both testis and somatic tissue compared with the control flies, where we did not detect any transcript (Fig. 1B). Then, by RT–qPCR, we detected an increase of approximately 60 times of the *TAHRE* transcript in comparison with the control flies (Fig. 1C).

It is known that *TAHRE*, as the other two elements of the HTT array, can be transcribed from both strands as it has two putative promoters at the 5′ and 3′ regions of the element ^29,30^. In order to determine if both strands derived from the 5′ and 3′ region of *TAHRE* are overexpressed by the action of the dCas9-VPR, we performed strand specific RT-qPCR analysis of the 3′ end of *TAHRE.* We showed that both strands are highly transcribed when transcription is induced with dCas9-VPR (Fig. 1D), indicating that the ectopically activation of *TAHRE* can operate for both strands. All together, these results indicate that dCas9-VPR can direct transcription of the heterochromatic *TAHRE*  element. Intriguingly, although we observed a very robust transcription of *TAHRE* in the testes detected by RT-qPCR, these transcripts could not be detected by in situ hybridization, suggesting that their half-lives are very short and that are rapidly degraded.

### Ectopic expression of the *Ubx* gene by directing the CRISPRa system to its promoter

**Figure 2:**
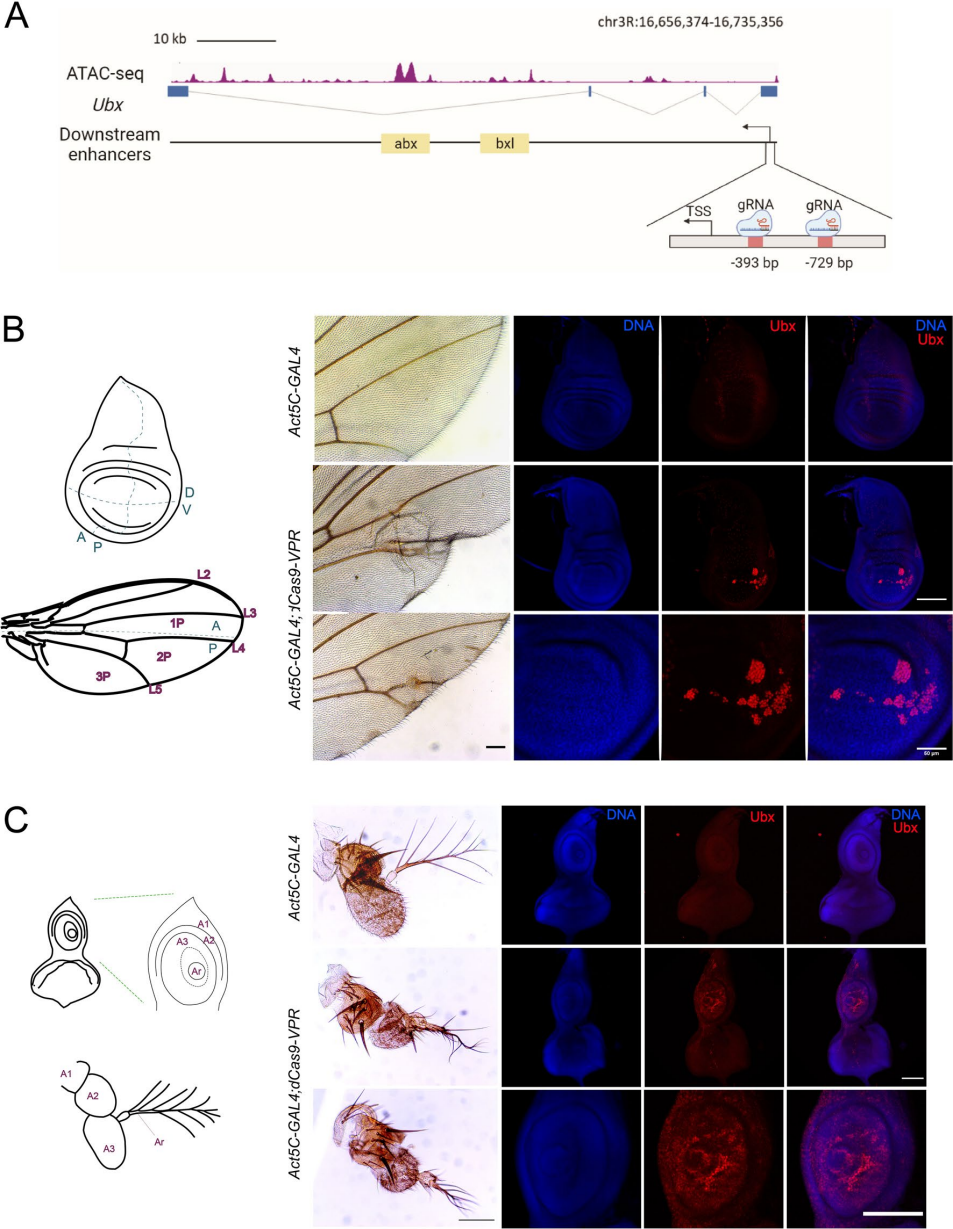
*Ubx* expression mediated by the dCas9-VPR directed to the *Ubx* promoter generates ectopic variegated expression causing homeotic transformations. (**A**) Map of the *Ubx* gene showing its genomic organization and the position of the gRNAs designed upstream of its promoter. The enhancers localized inside the body of the gene were mapped using previously reported ATAC-seq data^49^. The genome coordinates of the gRNAs positions are indicated. (**B**) Phenotypes generated by the ectopic expression of *Ubx* in adult wings and its corresponding imaginal disc comparing with control lacking dCas9-VPR activator. In the zoom of the pouch it is shown the variegated phenotype in *Act5C-GAL4;dCas9-VPR* disc. (**C**) Ectopic expression of *Ubx* in the eye-antenna disc and the transformed phenotypes from antennas to legs in adult organisms. The identification of Ubx was determined by immunostainings and DNA was stained with DAPI, Barr: 100 μm. The coordinates of the wing and the eye-antenna discs as well as the corresponding adult structures are indicated in the diagrams (for details see the text).

HOX genes can be considered a typical example of facultative heterochromatin because they are epigenetically spatiotemporally silenced by PRC1 and PRC2 complexes during *Drosophila* development^18,31,32^. To determine whether the CRISPRa system can activate the transcription of this type of gene in cells in which it is epigenetically silenced, we selected the HOX gene *Ubx. Ubx* controls the identity of thoracic segment 3 (T3) and the anterior part of abdominal segment 1 (A1)^33–35^. During larval stages, *Ubx* is expressed in the haltere and T3 metathoracic leg (T3 leg disc) imaginal discs. In cells where *Ubx* should not be expressed^36,37^, it is found as facultative heterochromatin enriched with the mark H3K27me3^38^. *Ubx* is transcribed from a single promoter, and its regulatory elements are extended by approximately 100 Kb (Fig. 2A)^39^.

Following the previous strategy, we expressed dCas9-VPR under the control of the *Act5C* driver in all fly tissues. The gRNAs were directed upstream of the transcription initiation site of the *Ubx* gene (Fig. 2A; Sup Table 1). Interestingly, although dCas9-VPR was expressed ubiquitously, we did not detect any reduction in viability. However, phenotypes that are typical of *Ubx* ectopic expression were observed in wings and antennae with a penetrance of 63% (Sup. Table 3). For instance, the mutant phenotype in adult wings is observed predominantly in the second (2P) and third (3P) posterior regions as well as in cubital (L4) and distal (L5) veins of the wing (Fig. 2B). Additionally, adult wings presented extra cell clusters in L4 and L5 and either extra or absence of veins, as well as extra bristles and patches of cells in 2P and 3P (Fig. 2B). In addition, mutant phenotypes consistent with an initial antenna-to-leg transformation were found. We observed the appearance of bristles in the arista and in the bulge of this structure (Fig. 2C). At the base of the arista, we also observed the appearance of extra bristles protruding from antennomere 3 (A3) (Fig. 2C). These phenotypes are consistent with those described for an antenna-to-leg transformation^40^ and references therein.

Following these results, we analyzed whether *Ubx*  is expressed in dCas9-VPR wing and eye-antenna imaginal discs given that these wild-type discs do not normally express this gene ^41,42^. In dCas9-VPR-expressing wing discs, patches of cells expressing *Ubx* were observed to be located mostly in the pouch along the dorsal-posterior area (Fig. 2B). For the eye-antenna imaginal disc, we detected the ectopic presence of *Ubx* predominantly in the antenna region and less so in the eye section (Fig. 2C). This pattern of *Ubx* expression in the wing and in the antenna-eye imaginal discs is consistent with the mutant phenotypes observed in the corresponding structures in the dCas9-VPR adults. These data suggest that the dCas9-VPR directed to the *Ubx* promoter can activate the ectopic expression of this gene in wing and antenna-eye imaginal discs, resulting in variegated expression, particularly of the posterior region in the wing disc.

Since the expression of dCas9-VPR was directed by a ubiquitous driver, we wondered if the ectopic expression of *Ubx* would increase by directing the expression of dCas9-VPR to a specific region of the wing disc. For this purpose, we used the specific wing *apterous* (*ap*-GAL4; to direct expression to all the wing disc pouch) and *MS1096* (to direct expression to the dorsal region of the wing disc pouch) drivers. As shown in Sup. Fig. 1, the effect on *Ubx* expression was similar to that observed using the actin driver. When the tubulin driver was used, which is stronger than *Act5C*, the transformation of antennae to legs was more dramatic (Sup. Fig. 1). Intriguingly, these results showed that there was no detectable expression of *Ubx* in other tissues, despite that the Act5C driver is ubiquitously expressed in all cells, suggesting that only in some specific tissues the *Ubx* ectopic expression may be conducted by the dCas9-VPR. Furthermore, similar results were obtained using the *αTub* and *wor* (neuroblasts specific) driver to identify if *Ubx* could be ectopically expressed in the nervous system in embryos (Sup. Fig. 1C). Nevertheless, not evident ectopic expression was found (Sup. Fig. 1C), supporting the idea that only in some particular cells the *Ubx* ectopic expression may be conducted by the dCas9-VPR system (see discussion).

In summary, the dCas9-VPR system, when directed to the *Ubx* promoter, can induce its variegated ectopic expression in some but not all imaginal discs, generating changes in the cell identity that are manifested in adult organs. In addition, ectopic *Ubx* expression in the wing imaginal disc preferentially occurs in specific regions, indicating that within the disc, there are cells that seem to be more permissive to derepress *Ubx* than others. These results indicate that the presence of an artificial transcriptional activator is sufficient to activate *Ubx* despite its normal silenced state in some cells of the wing and eye-antenna discs.

### Ectopic expression from the *Scr* promoter by the CRISPR/dCas9-VPR system

**Figure 3:**
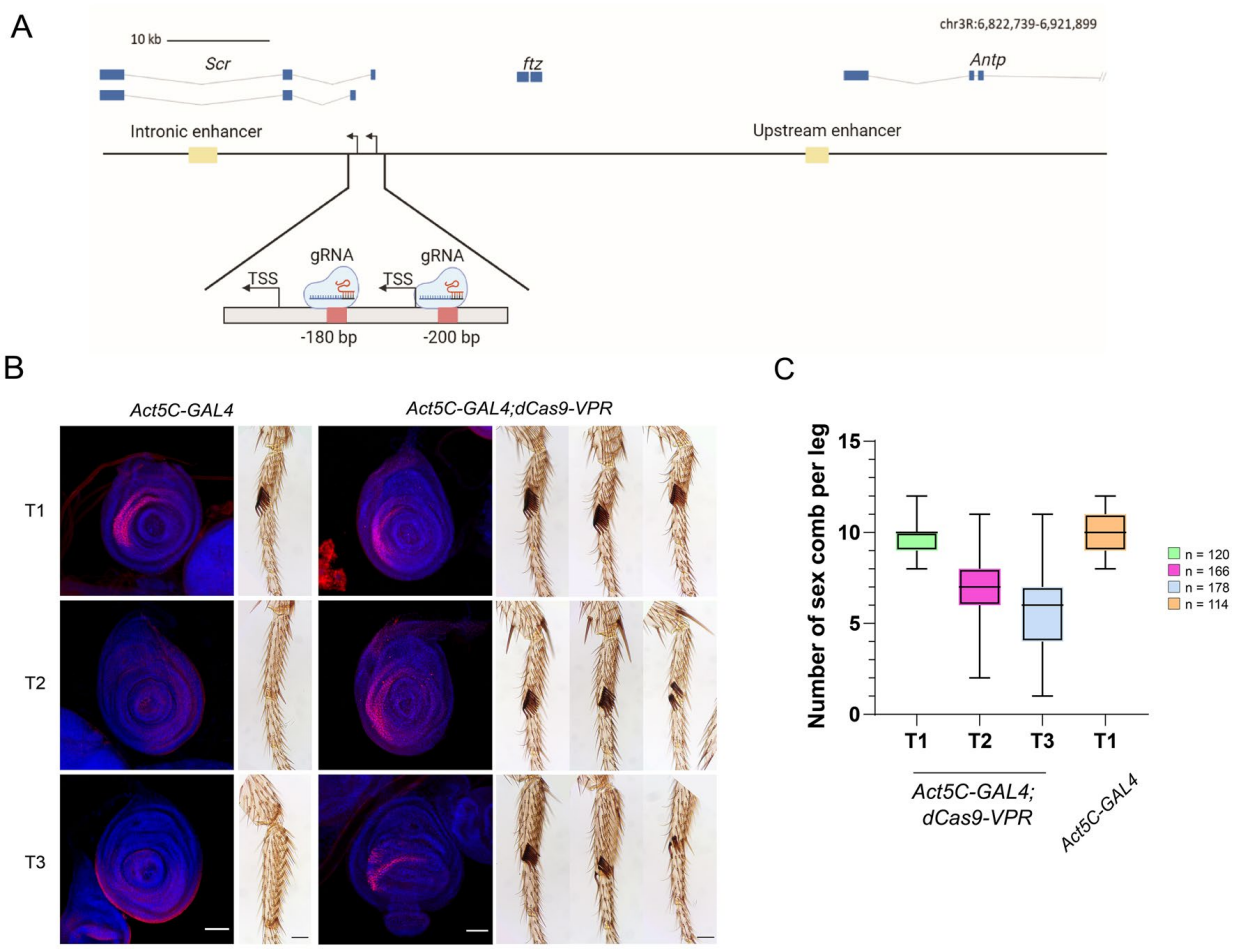
dCas9-VPR directed to the *Scr* promoter cause the transformation of the legs T2 and T3 into leg T1. (**A**) Map of the *Scr* gene showing its genomic organization, the position of enhancers that regulate *Scr,* as well as the position where the gRNAs were sent upstream of the *Scr* promoters. The genome coordinates of the gRNAs position is indicated. (**B**) Ectopic expression of *Scr* by immunostaining using the actin driver in the T2 and T3 legs discs (Barr: 50 μm.), along with the transformed phenotypes generated in adult legs. The red signal corresponds to Scr and the blue signal are the nuclei visualized by Hoechst-DNA staining. (**C**) Quantification of the expressivity indicated by the number of extra sex combs in the legs two and three (T2, T3) compared with control *Act5C-*GAL4 flies.

To extensively analyze the activation of transcription by the CRISPR/dCas9-VPR system in genes silenced by Pc, we also evaluated the *Sex comb reduced* (*Scr*) gene. *Scr is a* HOX gene involved in the differentiation of the labial and prothoracic segments^43^. Its ectopic phenotypes are identified in male adults by the presence of extra sex combs in the mesothoracic (T2) and metathoracic (T3) legs, similar to those present in the prothoracic leg (T1) in wild-type males^44^. Similar to *Ubx*, *Scr* is highly regulated during development, with specific enhancers localized upstream and inside the second intron, directing its expression at different locations and developmental stages^43^. The *Scr* gene encodes two isoforms that are transcribed from two overlapping promoters and, therefore, from two TSSs^44^. Two gRNAs were designed to bind to a region between the Polycomb Response Elements (PREs) 4/9, which are binding sites for PCR1 and PRC2 that cover the two TSSs^45^ (Fig. 3A; Sup. Table 1). Male adult flies expressing gRNAs and dCas9-VPR under the *Actin5C* driver had a strong fully penetrant gain-of-function *Scr* phenotype, showing the presence of ectopic sex combs in T2 and T3 legs (Fig. 3B-C). The expressivity (number of combs per leg) of the phenotype varies between different organisms (Fig. 3B), but 100% of males exhibit this homeotic transformation (Fig. 3C).

Next, we analyzed the *Scr* expression pattern in leg imaginal discs in males by immunostaining (Fig. 3B). As expected, we found ectopic expression of *Scr* in the T2 and T3 leg discs. In summary, the occupancy of dCas9-VPR of the *Scr* promoter region induces its expression in other leg discs where it is normally silenced, generating a typical Polycomb mutant phenotype ^32^. However, ectopic *Scr* expression besides the leg discs was not observed in other tissues.

### Targeting the dCas9-VPR to the *bxI* enhancer abolishes *Ubx* expression

**Figure 4:**
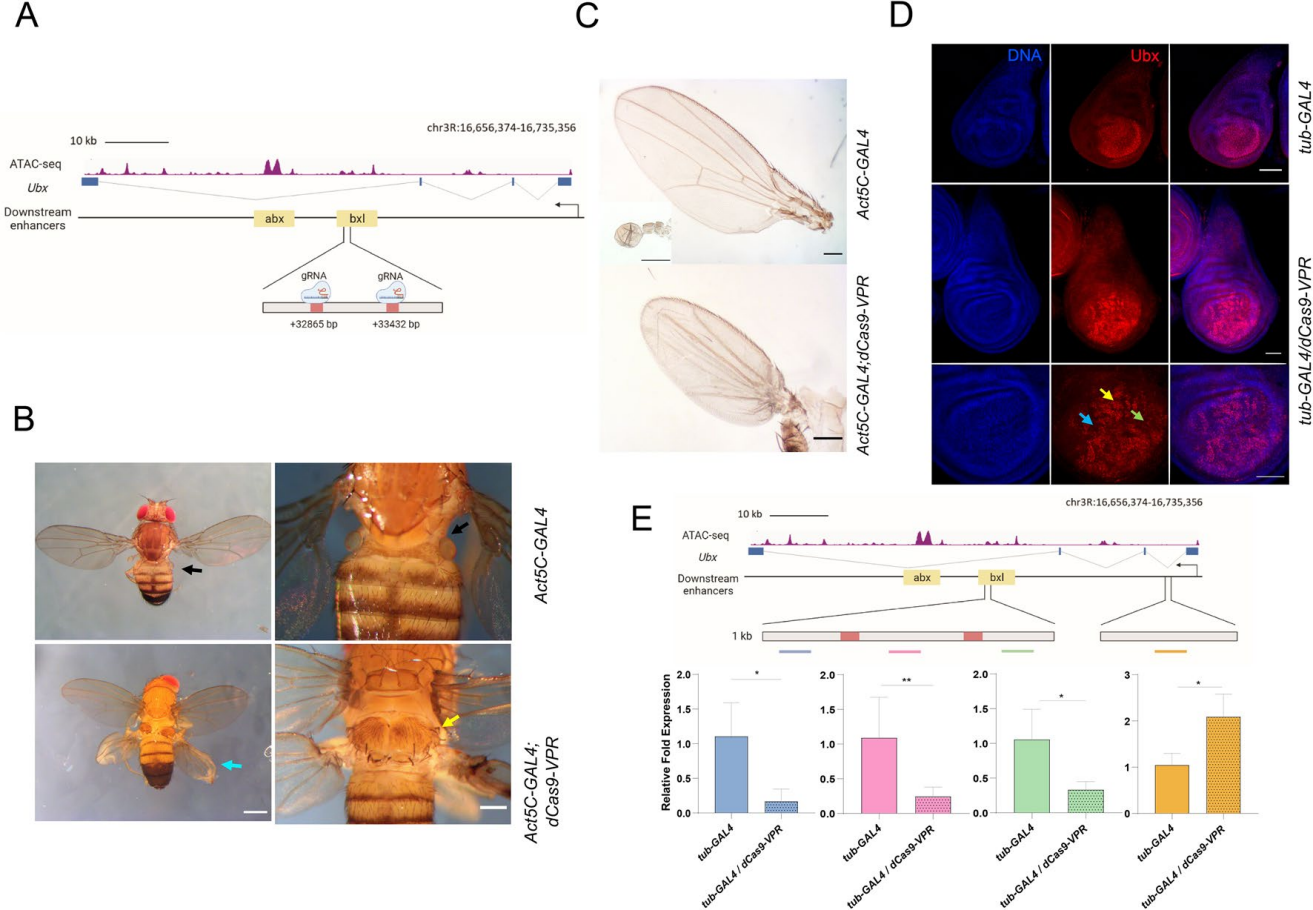
The presence of dCas9-VPR in the *bxI* enhancer generates a typical loss of function phenotype of *Ubx*. (**A**) Genomic organization of the *Ubx* locus showing the position of the gRNAs designed inside the *bxI* enhancer localized inside the third intron of the gen; the open chromatin regions were mapped using previously published ATAC-seq data^49^. The genome coordinates of the gRNAs position are indicated. (**B**) Homeotic transformations generated by the occupancy of the dCas9-VPR in the *bxI* enhancer. The arrows indicate the location of the halters in the *Act5C-GAL4* control fly (black) and the transformation of the metanotum (yellow) and the halteres to wings in the *Act5C-GAL4;dCas9-VPR* flies (blue). (**C**) Transformation of the halteres to wings comparing the size versus the control structures. (**D**) Immunostainings of Ubx in haltere discs when dCas9-VPR is sent to the *bxI* enhancer. The arrows mark cells that do not express Ubx (blue), express low levels of Ubx (green) and normal levels of Ubx (yellow). (**E**) RT-qPCR of the transcripts that surround the occupancy of the dCas9-VPR in the *bxI* enhancer and of the nascent *Ubx* transcript. At least three independent experiments were performed (see material and methods). Transcript levels of *rp49*  were used as a reference. The positions in the *Ubx* gene of the amplicons analyzed are indicated in the figure and its location is indicated in Sup. Table 2. *p < 0.05; **p < 0.01.

Recently, it has been reported that sending the CRISPRa system to enhancers can activate gene expression of the target gene^24^. Thus, we analyzed the effect of directing dCas9-VPR to enhancers of the HOX genes *Ubx* and *Scr*. *Ubx* is regulated during development by different enhancers that are tissue-specific^46^. The *anterobithorax* (*abx*) and *bithorax* (*bx*) regions contain several CREs that are in the third intron of *Ubx*, approximately 30 kb downstream of the TSS (Fig. 4A). These regions are functional in haltere imaginal discs^47^. The *bxI* region is responsible for part of the enhancer activity of *bx*^48^. Recently, CREs in this region have been dissected more precisely by chromatin accessibility assays^49,50^.

We directed the dCas9-VPR system to the *bxI* enhancer at 13,540 bp from the *abx* CRE^48,49^ based on public ATAC-seq data that show this region as  an open chromatin area in haltere cells^49^ (Fig. 4A). Intriguingly, transgenic flies with this construct presented a homeotic haltere-to-wing transformation as well as transformation of the metanotum corresponding to the anterior part of the third thoracic segment (T3a) to exhibit characteristics of the anterior part of the second thoracic segment (T2a) (Fig. 4B), consistent with *Ubx* loss-of-function phenotypes (Fig. 4B, C). The mutant phenotype had a penetrance of 63% (Sup. Table 4). Interestingly, no other defects were evident. This phenotype is generally present in adult flies when *Ubx* expression is reduced in the T3 thoracic segment, meaning that a reduction in *Ubx* activity in the haltere disc was generated by sending the dCas9-VPR transcriptional activator to the *bxI* enhancer. We analyzed Ubx levels by immunostaining the imaginal discs. First, we noticed that haltere discs with dCas9-VPR directed to the *bxI* showed a partial to total transformation to wing disc morphology (Fig. 4D). Additionally, we observed a variegated Ubx distribution in the distal region of the disc as well as a different distribution of Ubx compared to the wild type disc (Fig. 4D).

These results suggest that, contrary to what we expected, the binding of dCas9-VPR to the *bxI* enhancer generates an *Ubx* loss-of-function phenotype, although immunostaining indicates that Ubx levels and its distribution are only partially affected. This raises the question of whether the binding of dCas9-VPR in the intronic *bxI* affects enhancer activity or, as an alternative, blocks the elongation of RNPII, as has been reported when dCas9 is present downstream of the TSS of some genes^51–53^. Additionally, it is also possible that *Ubx* overexpression in haltere discs may generate a similar homeotic transformation, since *Ubx* negatively regulates its own expression^37^.

To determine which of these hypotheses is correct, we performed RT–qPCR experiments of the *bxI* enhancer region around the dCas9-VPR binding sites, as it is known that there is a correlation between enhancer activity and the transcription of the enhancer RNA (eRNA)^54–56^. We performed RT–qPCR of the three regions around the sites that recognize the gRNAs (Fig. 4E). Clearly, there was a decrease in the transcripts arising from this region of *bxI* (Fig. 4E), indicating that the presence of dCas9-VPR reduced transcription in that region. However, this result does not differentiate whether the synthetic transcription factor affects the function of the enhancer or prevents RNAPII elongation.

To answer this question, we quantified nascent pre-*Ubx*-RNA by RT–qPCR, targeting the first intron near the *Ubx* TSS (Fig. 4E). Intriguingly, the *Ubx* nascent transcript levels increased by twofold in haltere discs in which dCas9-VPR is targeted to *bxI*. However, *Ubx* transcription was not detected in the wing disc in the same flies (Sup. Fig. 2). These results indicate that the occupancy of dCas9-VPR in this CRE enhances its effect on *Ubx* transcription initiation only in the haltere disc. However, since this enhancer is within the body of the gene, we propose that the generation of the loss-of-function phenotype is because it blocks RNPII elongation in some cells.

### dCas9-VPR induces the overexpression of *Scr* when it is directed to the upstream enhancer

**Figure 5:**
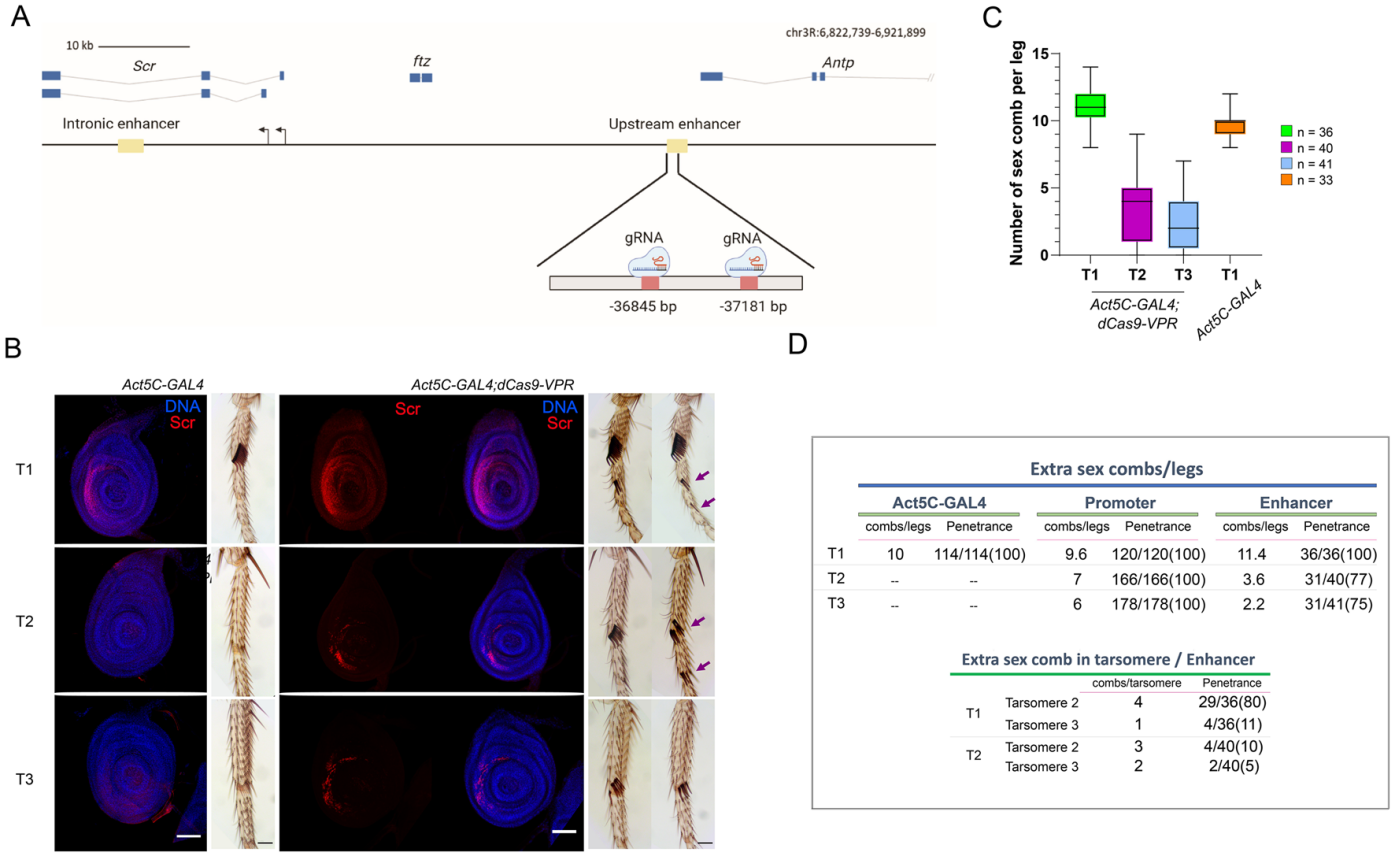
The occupancy of dCas9-VPR in the *Scr* upstream enhancer cause its ectopic expression and the generation of extra sex combs in T2 and T3 legs , as well as in tarsomeres 2 and 3**. (A**) Map of the *Scr* gene showing its genomic organization, the position of enhancers that regulate *Scr,* as well as the position where the gRNAs were directed in the upstream enhancer. The genome coordinates of the sgRNAs positions are indicated. (**B**) Extra sex combs phenotypes in male adult legs and the ectopic expression of *Scr* by immunostaining in leg discs. The arrows indicate the presence of extra sex combs in the tarsomeres T 2 and 3 in T1 and T2. Barr: 50 μm. (**C**) Quantification of the expressivity indicated by the number of extra sex combs in T2 and T3 compared with *Act5C*-GAL4 control flies. n = the number of legs analyzed. (**D**) Comparative table between the homeotic transformation phenotype between organisms in which the dCas9-VPR was sent the promoter region versus when it was sent to the upstream enhancer. The penetrance and the expressivity of the phenotypes are indicated.

The results presented up to this point with *Ubx* have shown that its effect on the induction of transcription by dCas9-VPR may not be the same when it is targeted to a promoter as it is when targeted to an enhancer. Thus, we tested the transcriptional effect of the dCas9-VPR system when directed to a nonintronic enhancer that is located far away from the gene it acted on, as is the case of the *Scr* upstream enhancer, *ScrE*^43^. This enhancer is at -33 Kb with respect to the *Scr* TSS of the two promoters, between the 3´ end of *fushi tarazu* (*ftz*) and the 3’ end of *Antennapedia* (*Antp*) (Fig. 5A). *ScrE* includes a subfragment of 439 bp that contains several putative binding sites for homeodomain TFs and that can direct the expression of *Scr* to specific regions in the T1 leg disc, including the primordia of the sensory organ^43^. We directed the dCas9-VPR approximately 1,200 bp upstream of this subfragment^43^ (Sup. Table 1). We found that these flies have ectopic sex combs in the T2 and T3 pairs of legs in the first tarsomere, indicating derepression of *Scr* in these discs (Fig. 5B), similar to the flies in which the dCas9-VPR was directed to the *Scr* promoter region (Fig. 1). However, the number of ectopic sex combs in flies where dCas9-VPR was directed to the *Scr* enhancer was significantly lower than that found in flies in which dCas9-VPR was sent to the *Scr* promoter region, as well as the penetrance of the phenotype (Fig. 5B–D). Intriguingly, in the T1 legs, sex combs are also present in the second and even in the third tarsomeres (Fig. 5B–D). Additionally, in some of the T2 legs, we found the same phenotype (Fig. 5C,D). In addition, the average number of sex combs in T1 flies was higher than that in wild type flies (Fig. 5C,D), suggesting an enhancement of the expression of *Scr* mediated by the action of dCas9-VPR on the *ScrE* enhancer in T1 (Fig. 5C,D).

We followed *Scr* expression through Scr immunostaining of leg discs and found that it was ectopically expressed in T2 and T3 discs. In agreement with the extra sex comb phenotype observed in adult male legs of this genotype, there was a lower number of cells in discs that expressed *Scr* than in discs from flies where dCas9-VPR was directed to the promoter (Fig. 3 vs. 5). Noticeably, Scr immunostaining was found in a variegated pattern, showing that not all cells responded similarly to *Scr* derepression. Additionally, we observed an increase in the number of cells that expressed *Scr* in the T1 disc, preferentially in the region that will give rise to tarsomeres 2 and 3, explaining the appearance of extra sex combs in these structures (Fig. 5B).

These results show that when dCas9-VPR is sent to the *Scr*-upstream enhancer *ScrE*, the ectopic expression of *Scr* in the T2 and T3 tarsi occurs at a lower level than when the synthetic transcription factor is sent to the promoter region, but there is also expression outside the disk domain where Scr is normally expressed.

## Discussion

In this work, we analyzed how a synthetic transcription factor, in this case dCas9-VPR, is capable of activating transcription in different types of chromatin. In the case of the telomeric element *TAHRE*, cataloged as constitutive heterochromatin and not transcribed in somatic tissues, the artificial activator was very efficient in inducing its transcription from regions corresponding to the 5’ and 3’ end of *TAHRE*. It is known that the HTT array is enriched in H3K9me3, which is recognized by the Rhino protein, an HP1 paralogous^57^. This combination of marks is indicative of constitutive heterochromatin. However, there are studies on the composition of histone marks using polytene chromosomes and immunostainings that have shown that this heterochromatin is rich in H3K9me3, and intriguingly also in H3K4me3^58^. Nevertheless, at that resolution is difficult to be able to say what are the decorations of the nucleosomes in *TAHRE*  when we send to dCas9-VPR to this element. Regardless of this, we demonstrated that dCas9-VPR induces robust transcription of *TAHRE in somatic cells and testis.* This is important since it has been reported that nucleosomes can prevent the binding of Cas9 to its target or affect its binding efficiency^59–63^. However, the results presented here clearly show that although *TAHRE* is part of the telomeric constitutive heterochromatin, dCas9-VPR is capable of binding to its target directed by gRNAs and activating RNPII-mediated transcription. Since dCas9-VPR does not cut DNA and contains three transcription activation domains, it could allow binding to its target in a more stable way even though the chromatin is formed as a nucleosome and, consequently, activate transcription. However, it cannot be ruled out that at some point in the cell cycle, the target DNA could be nucleosome-free, and dCas9-VPR efficiently binds to its target at this point, subsequently inhibiting nucleosome assembly in that region. In any case, dCas9-VPR induces robust transcription of *TAHRE*, suggesting that regions that are configured as constitutive heterochromatin can be transcriptionally activated if a transcription factor has the ability to bind to it.

Regarding the induction of ectopic transcriptional activation of the HOX *Ubx* and *Scr* genes by dCas9-VPR, some points are valuable to discuss. When dCas9-VPR was sent upstream of the *Ubx* promoter, ectopic expression in the wing and eye-antenna discs was variegated. This suggests that the binding of dCas9-VPR in the promoter region was not efficient in causing robust transcriptional activation in all cells or at the same time. Nevertheless, the clonal effect suggests that once *Ubx* is activated in a cell by means of the synthetic transcriptional activator, its expression is maintained in its daughter cells. It is also possible that the genome position to which the transcriptional activator was sent may not be optimal to activate transcription from this promoter, although it has been established that the binding site of the gRNAs approximately -400 bp from the TSS is adequate^26^. Intriguingly, something highly remarkable is that in the case of sending dCas9-VPR to the *Ubx* and *Scr* promoters, and to the *Scr* upstream enhancer, there is only ectopic expression in specific tissues and in a limited number of cells, even though the *Act5C * and *Tubulin*  drivers expressed GAL4 in all tissues during fly development. This suggests that in cells in which *Ubx* and *Scr* are not expressed, the chromatin is not permissive or lacks transcription factors that are required to efficiently promote their expression. In addition, it has been reported that in the promoters of some homeotic genes such as *Ubx* and *Abdominal-B* (*AbdB*), the RNPII is kept paused even when these genes are not expressed^64^. Therefore, it is possible that only in certain cells the RNPII is already present in these promoters and therefore the ectopic activation by the dCas9-VPR is facilitated. This aspect has to be taken into account when it is desired to use the CRISPRa system to ectopically express a gene. In any case, in some cells, the expression of *Ubx* was ectopically activated, bypassing the *Ubx* silencing exerted by the Pc group genes in these discs. On the other hand, ectopic *Scr* activation was more robust (100% penetrance, high expressivity), comparable to typical phenotypes in animals lacking PcG function. As mentioned above, *Scr* has two TSSs, and it is possible that the transcriptional activator would activate both promoters, although this has not been determined. In any case, dCas9-VPR  activated * Scr* expression in tissues in which it should be silenced by the PcG genes.

The phenotypes of animals where dCas9-VPR was sent to the *Ubx* or *Scr* enhancers were different from those where it was sent to the promoters. When dCas9-VPR was in the *Scr* upstream enhancer, *Scr* ectopic transcription was activated in T2 and T3 leg discs. However, its expression varied, with low penetrance and expressivity. Noticeably, *Scr* expression was extended toward the center of the disc, mainly in T1 and T2, causing the appearance of sexual combs in tarsomeres 2 and 3 of legs derived from these T1 and T2 discs.

The fact that the occupation of the synthetic activator in the *Scr* enhancer can activate transcription in the T2 and T3 discs suggests that the enhancer has almost all of the requirements necessary to activate the expression of *Scr* in these tissues and that the addition of a new transcription factor was sufficient to activate CRE. In support of this hypothesis, it has recently been reported in several organisms that the interaction between the enhancer and its target promoter occurs even though the enhancer is not active, both in the tissue where the target gene is expressed and in tissues where it is not^65–67^. This observation is relevant from an evolutionary point of view since mutations in Selector genes are known to be the cause of morphological differences between different species. For example, in other *Drosophila* species, the expression of *Scr,* unlike in *D. melanogaster*, causes the presence of sexual combs in tarsomeres 2 and 3^68–70^, similar to what we have observed when sending the dCas9-VPR to the *Scr* upstream enhancer.

When dCas9-VPR was sent to the *Ubx bxI* enhancer, it was able to increase *Ubx* transcription in the haltere disc, as determined by measuring the nascent *Ubx* transcript. However, a loss-of-function *Ubx* phenotype is observed in these flies. How is it possible that the effect of locating dCas9-VPR at the *bxI* enhancer is giving results at the same time of both *Ubx* transcriptional activation and repression? This seems to indicate that, as has been previously reported, the occupation of the dCas9-VPR downstream of the TSS, in this case at the intronic *bxI* enhancer, inhibits the passage of RNPII, causing a net effect of *Ubx* silencing. On the other hand, when dCas9-VPR occupies the *bxI* enhancer, it favors its interaction with the *Ubx* promoter, increasing the levels of transcriptional initiation. However, when RNPII reaches the downstream site where dCas9-VPR is positioned, it is stalled and aborted. Nevertheless, we do not know whether these two processes are occurring simultaneously or if the barrier action of dCas9-VPR on RNPII only occurs when the enhancer does not interact with the promoter. In support of this possibility, it has been reported that as transcription increases due to the action of activators in an enhancer, the distance between the promoter and the enhancer increases^71^. This could also explain why we observed an *Ubx* variegated expression pattern in haltere discs.

In conclusion, by using the synthetic transcription activator dCas9-VPR, we have shown that it is capable of activating transcription by RNPII, both in regions classified as constitutive (the *TAHRE* element) and facultative (the HOX genes) heterochromatin regardless of their silenced state. Likewise, the effect on transcriptional activation by sending this artificial activator to a promoter or to an enhancer of the same gene can generate different transcriptional responses in terms of the strength of an ectopic phenotype, as was the case for *Scr*. Surprisingly, the presence of dCas9-VPR in an enhancer within the body of *Ubx* can simultaneously increase the degree of transcription initiation and block the passage of RNPII in the same gene when located 30 kb from the TSS. All these findings show that since transcription is a stochastic and plastic mechanism, synthetic transcriptional activators in complex organisms are incorporated in a specific manner into each gene or regulatory region.

## Methods

### gRNAs cloning

Double guides, directed to the different targets of the *Ubx* and *Scr* genes and the retroelement *TAHRE*, were cloned into pCFD4-U6:1-U6:3 (Addgene plasmid #49,411. tandem expression vector) as previously reported^72^. For Gibson Assembly protocol, we used Gibson Assembly Master Mix de New England BioLabs. All guide sequences were designed using the Benchling platform^73^ and Breaking-Cas tool^74^. Guide sequences are available in Supplementary Table 1.

### Transgenic flies

The double gRNA-plasmids in pCFD4 were integrated into the fly genome at the attP40 integration site on the second chromosome with the phiC31 transformation method by the company BestGene.

Flies with genotype *gRNA/CyO;MKRS,Sb/TM6B,Tb,Hu* with each gRNA sequences directed to *TAHRE*, *Ubx* or *Scr* or their corresponding enhancers, were crossed with flies *W;If/CyO,UAS:dCas9-VPR/TM6B,Tb,Hu* (donated by Dr. Norbert Perrimon) in order to found the families with genotype *sgRNA/CyO;UAS:dCas9-VPR/TM6B,Tb,Hu.* Finally, we crossed these flies with the corresponding GAL4 driver, accordingly to the experiment. We used *Act5C*-GAL4 driver (Bloomington Stock Center, #4414) and *Tub*-GAL4 (Bloomington Stock Center, #5138) for ubiquitous expression in *TAHRE*, *Scr* and *Ubx* lines respectively, and specially with *Ubx* promoter lines: *MS1096-GAL4/y*, and *ap* > *GFP-GAL4/CyO,Tb;* +*/*+ in order to drive expression to the haltere imaginal disc. Fly crosses were conducted at 25 °C, unless for *bxI* line, which were performed at 28/18 °C each 12 h.

### Cuticle treatment

All structures dissected, including antennae, halteres, legs, and wings, were collected from adult fly which were boiled with KOH solution (10% v/v) in a water bath for 5 min. Then, were again boiled in sterile distilled water to remove KOH during 5 min. The structures were dissected in ethanol and mounted in glycerol 50%.

All cuticles dissected had the genotype: *sgRNA/Act5C-GAL4;UAS:dCas9-VPR/*+ with the g-RNA directed to *Ubx* and *Scr* promoters and enhancers as it may apply. As control phenotype we used the corresponding sister lines product of the cross with *Act5C*-GAL4 driver, which genotypes were *sgRNA/CyO;UAS:dCas9-VPR/*+ or *Act5C-GAL4/CyO;UAS:dCAs9-VPR/*+.

All cuticles were documented in a Nikon Eclipse E600 upright microscope with a digital camera with Aptina CMOS sensor 5.1 MP, KPA.

### Immunohistochemistry

Wandering-stage third instar larval were dissected in cold PBS 1X and fixed with 4% paraformaldehyde in PBS at room temperature for 30 min. Subsequently, larvae were washed 3 times with PBST (1X PBS and 0.2% Triton) during 15 min each wash. Then, larvae were blocked with BBT (1X PBS, 0.1% BSA and 250 mM NaCl) at 4 °C for 1 h. Afterwards, the sample was incubated with the primary antibody overnight. After that the larvae were washed again 3 times for 20 min with PBST, the secondary antibody and DAPI was added, and incubated for 2 h at room temperature, and washed 3 times with PBST for 20 min. Finally, the PBST was removed to add the mounting medium and thus extract the imaginal discs for later observation.

In the case of haltere, wing, and eye-antennae imaginal discs for *Ubx* promoter experiments, we analyzed flies with genotype *Ubx-gRNA/Act5C-GAL4;UAS:dCas9-VPR/*+ and lines *Ubx-gRNA/CyO;UAS:dCas9-VPR/*+ or *Act5C-GAL4/CyO;UAS:dCAs9-VPR/*+ as siblings lines control. In the case of *bxI* enhancer experiments, we used the haltere imaginal discs with the genotype *bxI-gRNA/CyO;UAS:dCas9-VPR/αTub-GAL4* for CRISPRa fenotype and *bxI-gRNA/CyO;UAS:dCas9-VPR/TM6B,Tb,Hu* as the control. The change to *αTub* driver corresponds to the use TM6B, Tb balancer for an easier identification of each genotype during third larval stage and as substitute for another ubiquitous driver similar to Act5C.. Finally, for both *Scr* experiments, promotor (*Scr-*gRNA) and enhancer (*ScrE-gRNA*), we performed the immunohistochemistry of leg imaginal discs with genotype *gRNA/Act5C-GAL4;UAS:dCas9-VPR/*+ and lines *gRNA/CyO;UAS:dCas9-VPR/*+  or *Act5C-GAL4/CyO;UAS:dCAs9-VPR/*+ as siblings lines control.

Antibodies used were mouse monoclonal antibody Ubx (Ubx FP3.38 DSHB; 1:50), mouse monoclonal antibody Scr (anti-Scr 6H4.1 DSHB; 1:50). As secondary antibody, we used Goat anti-mouse IgG Alexa Fluor 568 (A-11004; Invitrogen) at 1:200 and DAPI concentration 1 ng/μL.

Samples were imaged on Olympus FV1000 Multi-photonic Inverted or Olympus FV1000 Confocal Upright microscopes, equipped with a UPLSAPO objective 20X NA: 0.75 AND 60X NA: 1.1. Images were processed using ImageJ (Fiji) and Inkscape vector graphics editor software.

### Quantitative PCR assay

Adult specimens with genotype *gRNA/Act5C-GAL4;UAS:dCas9-VPR/*+ expressing gRNAs for *TAHRE* as CRISPRa lines and the resulting siblings lines from the cross as control were used. Testis and the corresponding carcasses of males without testis (somatic tissue) were analized. Haltere and wing imaginal discs for *bxI* fly lines with the genotype *bxI-gRNA/CyO;UAS:dCas9-VPR/αTub-GAL4* were used. The corresponding siblings lines obtained from the cross were used as controls.

The RNA was extracted and purified with TRIzol Reagent following the manufacturer’s protocol. The isolated RNA was used for the synthesis of cDNA, following the protocol for the M-MLV Reverse Transcriptase enzyme (Invitrogen). End-point PCR amplification of the cDNA was carried out to determine the presence of TAHRE transcripts using Taq polymerase. RP49 was used as a reference gene. For the stand-specific experiments, specific primers to sense and antisense TAHRE transcript were used for the cDNA synthesis, as well as the reverse primer of *rp49*  to use it as a reference gene.

The RT-qPCR experiments were conducted using LightCycler FastStart DNA Master PLUS SYBR Green I (Roche) for *TAHRE* experiments and Maxima SYBR Green/ROX qPCR Master Mix (Thermo Scientific) for the *bxI* enhancer experiments in a Roche Real Time PCR LightCycler 1.5. The threshold cycle (C_T_ or 2^−∆∆CT^) method^75^ was used to calculate the fold-change for transcript relative quantification. At least three independent biological replicates were analyzed in each case. Statistical analyzes (student t and One-way ANOVA tests) were performed using the GraphPad Prism 8 software. The primers used for RT-qPCR experiments for each target are listed in suppl. Table 2.

### Public ATAC-seq data analysis

ATAC-seq data from *Drosophila melanogaster* haltere were previously published^49^. Raw data was downloaded from the GEO database (GSE166714). Fastq files were aligned to the dm6 genome using Bowtie2 software and peak calling was performed using MACS3. BigWig files were processed using Deeptools library and finally were visualized using the IGV software.

## Supplementary Information

Supplementary Information 1.

Supplementary Information 2.

Supplementary Information 3.

Supplementary Information 4.

## Data Availability

All data generated or analysed during this study are included in this published article [and its supplementary information files]. The CRISPRa flies generated in this work are available under request to Dr. Mario Zurita mario.zurita@ibt.unam.mx.
