# Supplementary material for: Different transcriptional responses by the CRISPRa system in distinct types of heterochromatin in Drosophila melanogaster
Source: Sci Rep. 2022 Jul 9;12:11702. doi: 10.1038/s41598-022-15944-7 (PMC9271074; doi:10.1038/s41598-022-15944-7)
Supplement: Supplementary file 1 — Supplementary Information 1. [file 41598_2022_15944_MOESM1_ESM.docx]

**Supporting information**

**Sup. Fig**. 1. ***Ubx* ectopic expression by the Cas9-VPR directed to its promoter using different GAL4-drivers.**

A) Expression of *Ubx* in the wing disc modulated by the wing specific drivers *apterus* and MS1096. Note that the *Ubx* expression (Red) preferentially occurs in the posterior region of the disc in both cases. B) Transformation of antennas to legs using the *α*Tub-GAL4 driver. Note that the expressivity of the phenotype is more dramatic than when the *Actin5C*-driver is used. The corresponding genotypes are indicated in the figure. C) Expression of *Ubx* in drosophila embryo modulated by neuroblast specific driver *worniu.* *Ubx* expression (Green) occurs in pasasegments 6 to 12, and using this driver we even not observed ectopic expression along the central nervous system in the embryo. Scale barr: 100 µm.

**Sup. Fig**. 2. Loss of function phenotypes and quantification of the expression of *Ubx* in wing discs by sending the dCas9 to the *bxI* enhancer using the tubulin driver.

A) Examples of pharates that cannot hatch when dCas9-VPR is sent to the *bxI* enhancer using the tubulin-GAL4 driver, showing almost complete transformation of haleteres to wings.

B). Quantitative qRT-PCR analysis of the *Ubx* nascent transcript in wing discs of flies in which dCas9-VPR is sent to the *bxI* enhancer. Note that that unlike the case of the haltere disc, the Cas9-VPR does not enhance the *Ubx* transcription in this tissue.

**Table 1.** Sequence of the different sgRNA´s used in this work.

| Target | sgRNA sequence | PAM | Distance from TSS | On target score | Offtarget score |
| --- | --- | --- | --- | --- | --- |
| *Ubx* TSS | CCATATTCTAGCACAAAGAT | TGG | -393 | NA | NA |
|  | ATATTATAATTCGTTTTTAA | AGG | -729 | NA | NA |
| *Ubx bxI* | AGGACACGGAGCCACACGAT | CGG | +32865 | 65.1 | 49.6 |
|  | GATTGGGCTCCGGATCGCGA | GGG | +33432 | 65.9 | 48.3 |
| *Ubx bxd* | ACGAACGACAGTTATGGCGA | CGG | -29676 | 67.5 | 49.9 |
|  | AGCAGTAGCATTTCGCACTG | GGG | -29927 | 67.4 | 49.4 |
| *Scr* TSS | TATCCGCTATGGGGAGACCA | TGG | +200 | 64.4 | 49.6 |
|  | AGTCTTGATTTATGCGACGA | GGG | -180 | 64.9 | 49.8 |
| *ScrE* | TTGCAAAATGAGCTGGAGAG | AGG | -36845 | 66.8 | 48.0 |
|  | TGCACACACCTGTACTCAGG | CGG | -37181 | 64.2 | 49.9 |
| *TAHRE** | AAGCTGCCGCCATAACCAAA | AGG | -167(AUG)* | NA | NA |
|  | GGCAAAATAAATTGTGGATG | CGG | -503(AUG)*^±^ | NA | NA |

*In the case of *TAHRE* the distance is regarding the possible start codon for the translation of the Gag gene.

^±^This sgRNA can be also found united to 3’ UTR segment in TAHRE rTE.

**Table 2**. Primers used for RT-qPCR for each region analyzed

| Gene Targeted | Forward Primer | Reverse Primer | Alignment temperature | Ref |
| --- | --- | --- | --- | --- |
| *Rp49* | TCAAGATGACCATCCGCCCA | GTTCTCTTGAGAACGCAGGC | 60°C | ^72^ |
| *TAHRE* Gag ORF | CTTCCCCTCCGCTCTCATC | CCTAGATCTGCATTTGTATTAGTAGCTG | 65°C | ^73^ |
| *TAHRE* 3’UTR | CCATAGTCAGCATCTTTCTGTGGA | TCGCATCACTTCGTCATGATCAG | 58°C | This work |
| *Ubx* promoter | GGTCTGTTCTGCCTGCTTTC | GCCGTCTCTGCTCCAATTAAC | 59°C | This work |
| *bxI* enhancer Downstream | TACATATAACGACGACTTCC | TAGCAGCAGGTAAATGCGGC | 50°C | This work |
| *bxI* enhancer Middle | GAAGGCTGGGTGGAATAATT | GGGAACCGTTTTTTATGTGT | 60°C | This work |
| *bxI* enhancer Upstream | TAAGCCCAAACACTCGACTC | CCTGTGGAATGTAAATGTTG | 58°C | This work |
| *Ubx* first intron | GGTCTGTTCTGCCTGCTTTC | GCCGTCTCTGCTCCAATTAAC | 59°C | This work |

**Table 3.** Viability and penetrance for homeotic transformations in *Ubx TSS* fly lines.

|  | Viability | Homeotic transformation |
| --- | --- | --- |
| ***Ubx-sgRNA/Act5C-GAL4;UAS:dCas9-VPR/+*** | 59/59 (100) | 37/59 (63%) |
| *sgRNA/Act5C-GAL4;MKRS/+* | 58/59 (98) | 0 |
| *Act5C-GAL4/CyO;UAS:dCAs9-VPR/+* | 53/59 (90) | 0 |
| *sgRNA/CyO; UAS:dCas9-VPR/+* | 53/59 (90) | 0 |
| *Act5C-GAL4/CyO;MKRS/+* | 57/59 (97) | 0 |
| *sgRNA/CyO; MKRS/+* | 57/59 (97) | 0 |

**Table 4**. Viability and penetrance for homeotic transformations in *Ubx-bxI* organisms.

| Experiment RT | Viability | Homeotic transformation |
| --- | --- | --- |
| ***bxI-sgRNA/Act5C-GAL4;UAS:dCas9-VPR/+*** | 94/96 (97.4) | 59/94 (63%) |
| *sgRNA/Act5C-GAL4;MKRS/+* | 95/96 (98) | 0 |
| *Act5C-GAL4/CyO;UAS:dCAs9-VPR/+* | 88/96 (91.7) | 0 |
| *sgRNA/CyO; UAS:dCas9-VPR/+* | 88/96 (91.7) | 0 |
| *Act5C-GAL4/CyO;MKRS/+* | 96/96 (100) | 0 |
| *sgRNA/CyO; MKRS/+* | 96/96 (100) | 0 |

| Experiment 28/18°C | Viability | Homeotic transformation |
| --- | --- | --- |
| ***bxI-sgRNA/Act5C-GAL4;UAS:dCas9-VPR/+*** | 27/178 (15.2) | 13/27 (48.1%) |
| *sgRNA/Act5C-GAL4;MKRS/+* | 178/178 (100) | 0 |
| *Act5C-GAL4/CyO;UAS:dCAs9-VPR/+* | 117/178 (66) | 0 |
| *sgRNA/CyO; UAS:dCas9-VPR/+* | 117/178 (66) | 0 |
| *Act5C-GAL4/CyO;MKRS/+* | 131/178 (73.8) | 0 |
| *sgRNA/CyO; MKRS/+* | 131/178 (73.8) | 0 |
