# Supplementary figures and images for: Different transcriptional responses by the CRISPRa system in distinct types of heterochromatin in Drosophila melanogaster
Source: Sci Rep. 2022 Jul 9;12:11702. doi: 10.1038/s41598-022-15944-7 (PMC9271074; doi:10.1038/s41598-022-15944-7)

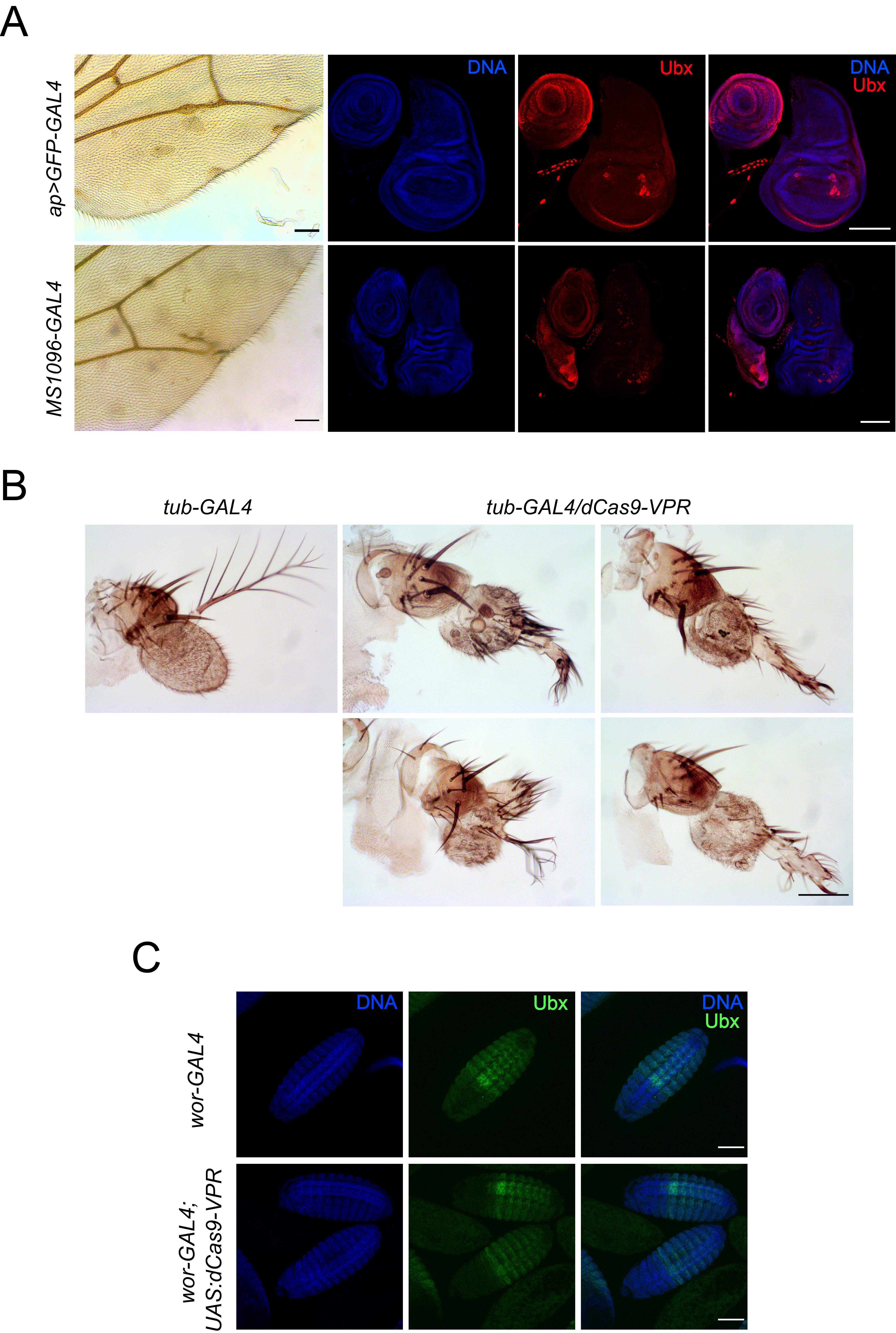

Supplement: Supplementary file 2 — Supplementary Information 2. [file 41598_2022_15944_MOESM2_ESM.jpg]

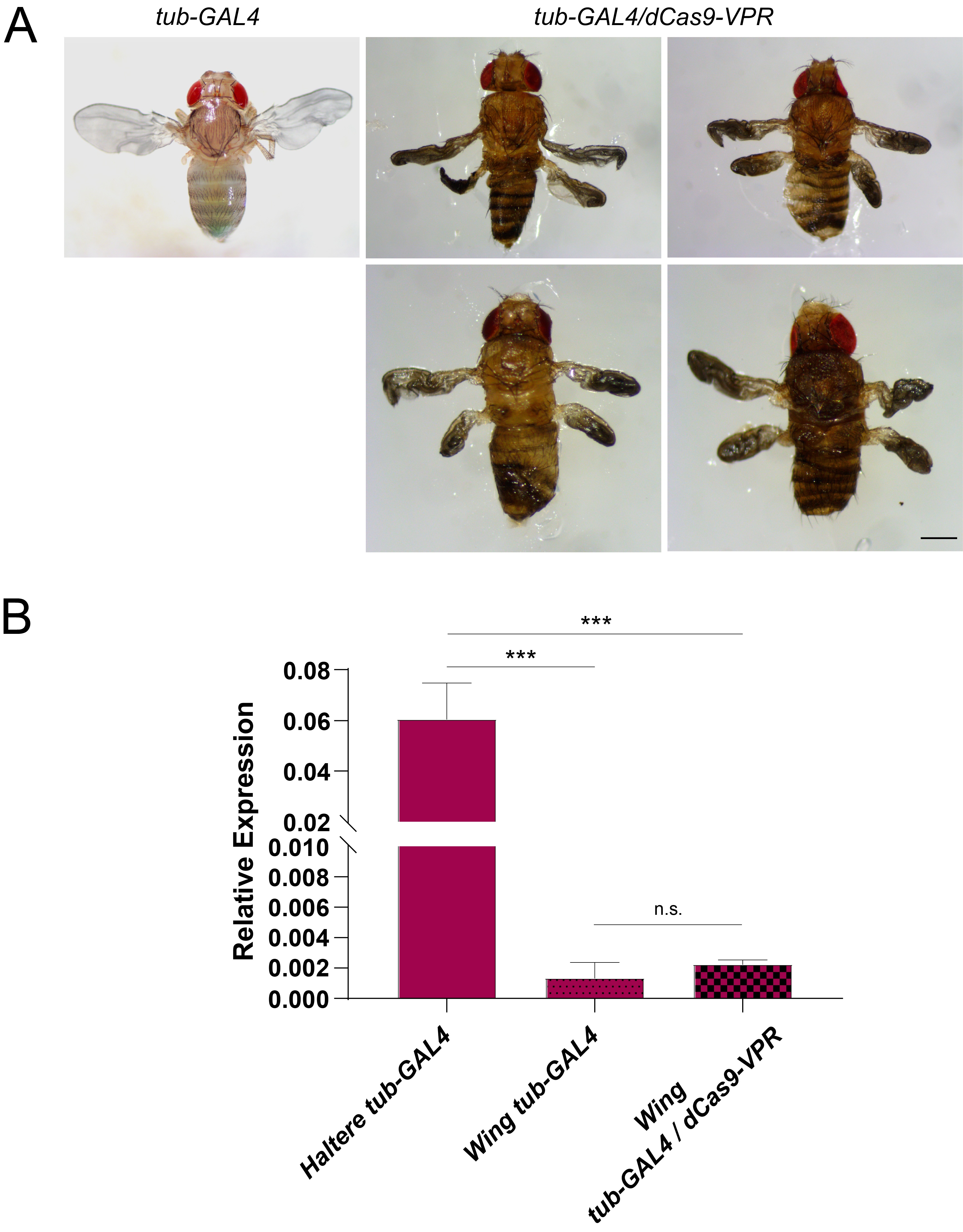

Supplement: Supplementary file 3 — Supplementary Information 3. [file 41598_2022_15944_MOESM3_ESM.jpg]
