# Supplementary material for: Different transcriptional responses by the CRISPRa system in distinct types of heterochromatin in Drosophila melanogaster
Source: Sci Rep. 2022 Jul 9;12:11702. doi: 10.1038/s41598-022-15944-7 (PMC9271074; doi:10.1038/s41598-022-15944-7)
Supplement: Supplementary file 4 — Supplementary Information 4. [file 41598_2022_15944_MOESM4_ESM.pdf]

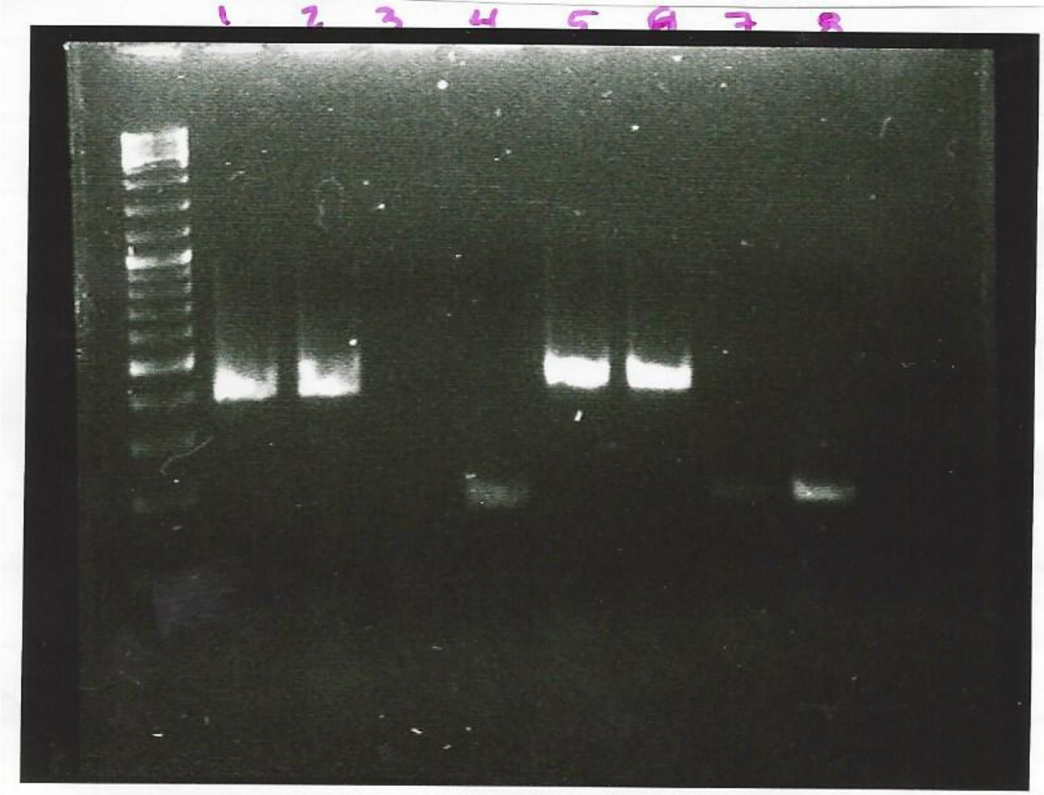

1:- cDNA Testis Control } + RP49
2:- cDNA Testis Mutante
3:- cDNA ~~Cuerpo~~ Testis Control } + TAHRE gag
4:- cDNA Testis Mutante
5:- cDNA Cuerpo control } RP49
6:- cDNA Cuerpo Mutante
7:- cDNA Cuerpo Control } TAHRE gag
8:- cDNA Cuerpo Mutante
9:-
